# First transcriptome analysis of bryozoan *Fredericella sultana*, the primary host of myxozoan parasite* Tetracapsuloides bryosalmonae*

**DOI:** 10.7717/peerj.9027

**Published:** 2020-04-28

**Authors:** Gokhlesh Kumar, Reinhard Ertl, Jerri L. Bartholomew, Mansour El-Matbouli

**Affiliations:** 1 Clinical Division of Fish Medicine, University of Veterinary Medicine Vienna, Vienna, Austria; 2 VetCore Facility, University of Veterinary Medicine, Vienna, Vienna, Austria; 3 Department of Microbiology, Oregon State University, Corvallis, OR, United States of America

**Keywords:** Bryozoan, Fredericella sultana, Transcriptome

## Abstract

Bryozoans are aquatic invertebrate moss animals that are found worldwide. *Fredericella sultana* is a freshwater bryozoan and is the most common primary host of myxozoan parasite,* Tetracapsuloides bryosalmonae*. However, limited genomic resources are available for this bryozoan, which hampers investigations into the molecular mechanisms of host-parasite interactions. To better understand these interactions, there is a need to build a transcriptome dataset of *F. sultana*, for functional genomics analysis by large-scale RNA sequencing. Total RNA was extracted from zooids of *F. sultana* cultivated under controlled laboratory conditions. cDNA libraries were prepared and were analyzed by the Illumina paired-ends sequencing. The sequencing data were used for de novo transcriptome assembly and functional annotation. Approximately 118 million clean reads were obtained, and assembled into 85,544 contigs with an average length of 852 bp, an N50 of 1,085 bp, and an average GC content 51.4%. A total of 23,978 (28%) contigs were annotated using BLASTX analysis. Of these transcripts, 4,400 contigs had highest similarity to brachiopod species *Lingula anatina*. Based on Gene ontology (GO) annotation, the most highly scored categories of biological process were categorized into cellular process (27%), metabolic process (24%), and biological regulation (8%) in the transcriptome of *F. sultana*. This study gives first insights into the transcriptome of *F. sultana* and provides comprehensive genetic resources for the species. We believe that the transcriptome of *F. sultana* will serve as a useful genomic dataset to accelerate research of functional genomics and will help facilitate whole genome sequencing and annotation. Candidate genes potentially involved in growth, proteolysis, and stress/immunity-response were identified, and are worthy of further investigation.

## Introduction

Members of the Phylum Bryozoa are generally small, sessile invertebrates that live on submerged surfaces, with most freshwater bryozoans grouped in the class Phylactolaemata (Wood et al., 1998). As filter feeders, they capture small particles e.g., unicellular algae with ciliated tentacles. Bryozoans are typically colonial, consisting of several hundred connected but individual zooids, with each zooid having its own independent tentacular lophophore mouth, gut, muscle, nervous, and reproductive systems (Wood et al., 1998). All bryozoans are hermaphrodites, but in some species separate male and female zooids are found in a single colony. Bryozoans reproduce both sexually (larvae) and asexually through budding and forming encapsulated, seed-like statoblasts, which remain dormant during unfavourable conditions and when conditions are favourable, the statoblasts germinate to form a new colony. The colonies grow rapidly during spring and form tubular and branching colonies by summer (Wood & Okamura, 2005; Abd-Elfattah, El-Matbouli & Kumar, 2017).

*Fredericella sultana* (Blumenbach 1779*)* is a cosmopolitan freshwater bryozoan found throughout Europe, North America, Asia, Australia, and New Zealand. The colonies are attached to submerged surfaces, typically pieces of wood or tree roots (Okamura et al., 2011). *F. sultana* is the most common invertebrate bryozoan host of myxozoan parasite *Tetracapsuloides bryosalmonae*, the causative agent of proliferative kidney disease, a disease of salmonids responsible for economically important losses in farmed trout (Okamura & Wood, 2002). *T. bryosalmonae* develops and proliferates in the body cavity of *F. sultana*. Infected zooids release parasite spores to the surrounding water (Feist et al., 2001; Okamura & Wood, 2002). Infected zooids that migrate from deteriorating bryozoan colonies to new habitats can contribute to further spread of *T. bryosalmonae* (Gorgoglione, Kotob & El-Matbouli, 2016). Additionally, statoblasts produced by infected colonies are responsible for vertical transmission of *T. bryosalmonae* (Abd-Elfattah et al., 2014). Hence, bryozoans are a potential dispersal source for *T. bryosalmonae.*

Developments in next-generation sequencing and assembly algorithm have made it possible to build the entire transcriptome of a particular species of interest (Sudhagar, Kumar & El-Matbouli, 2018). The transcriptome of the marine bryozoan *Bugula neritina* has been sequenced and functionally analyzed, which clearly demonstrated its utility for various stages of metamorphosis and neuropeptidome studies (Wang et al., 2010; Wong et al., 2014; Wong et al., 2016). However, little is known about transcripts of freshwater bryozoans. To date, 542 transcripts of *F. sultana* have been deposited in the GenBank database. This limited number of transcripts is insufficient for understanding the biology of freshwater bryozoan. Therefore, conduct a de novo assembly of the transcriptome is an important step to annotate, assign functional attributes to the assembled sequences, and set the stages for further molecular studies such as ecology, evolution, behavior, nutritional and host-parasite interaction of this highly important bryozoan species.

In this study, we aimed to provide the first transcriptome of freshwater invertebrate bryozoan *F. sultana* using the Illumina NextSeq550 sequencing platform, and to present a comprehensive analysis of the de novo transcriptome sequencing result.

## Materials & Methods

### Collection of *F. sultana* zooids

**Figure 1:**
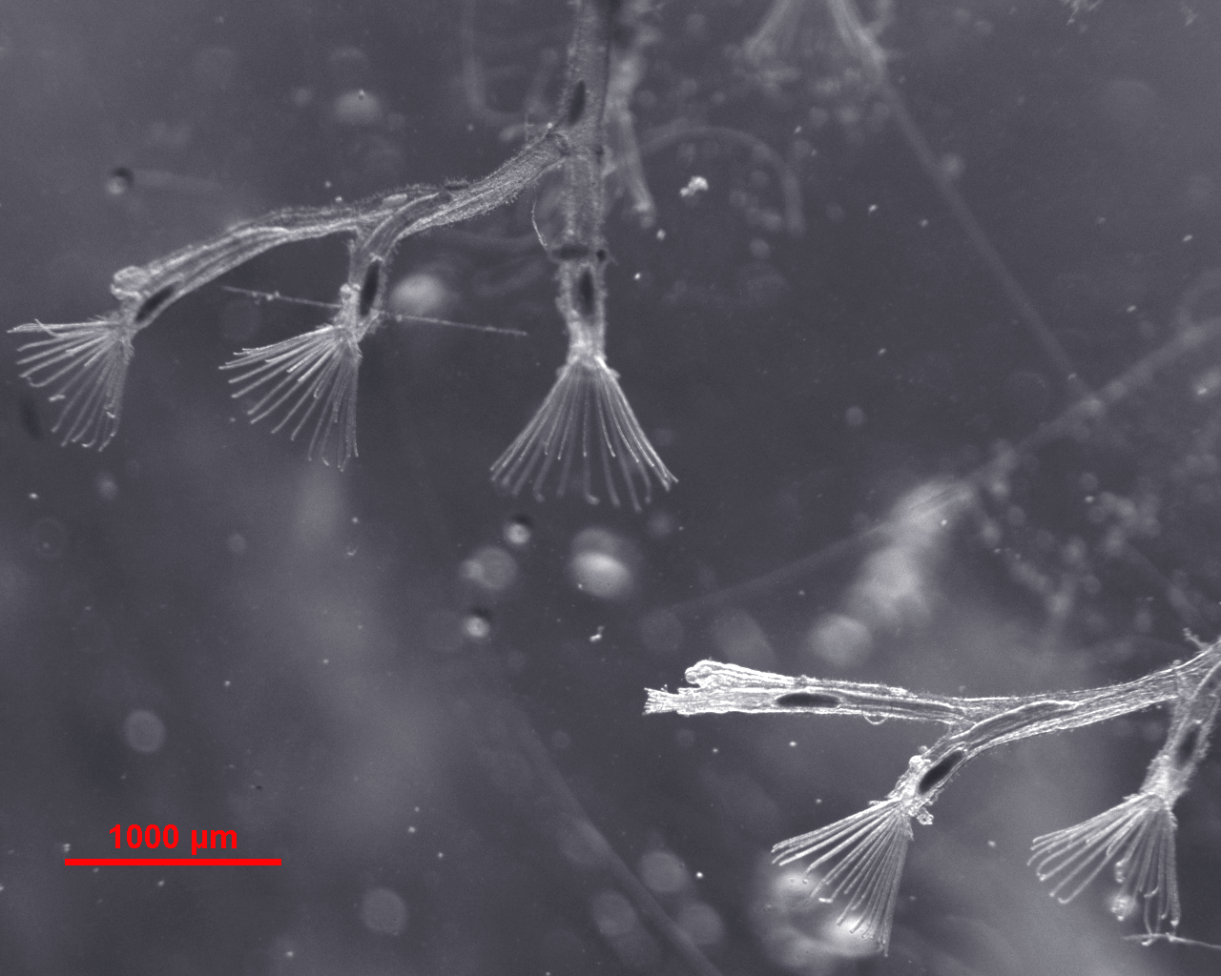
Bryozoan *Fredericella sultana* colony. Clean and transparent laboratory-cultured *F. sultana* with zooids showing tentacles.

The colony of *F. sultana* was reared in our laboratory after germination of specific pathogen-free statoblasts. The colony was maintained in an aquarium at 18 ± 1 °C according to Kumar et al. (2013). Two days before sampling, colonies were not fed with algae species (*Cryptomonas* and *Synechococcus*) to completely release the digested food particles from the gut of zooids. Additionally, colonies were replaced with clean dechlorinated tap water twice a day and were maintained under the clean culture system. Clean and transparent zooids (Fig. 1) were separated from the colony using needles and forceps. Zooids were washed gently in a Petri plate by replacing the water then immediately preserved in *RNAlater* and stored at −80 °C. Each sampled zooid includes a completely developed cystid body wall, and polypide (the lophophore and digestive tract).

### RNA extraction

Individual zooid samples (*n* = 3) were suspended in 600 µl of RLT buffer (Qiagen, Hilden, Germany) containing 6 µl of β-mercaptoethanol. Each sample was homogenized using the TissueLyser II (Qiagen) in the presence of steel beads for 2 min at 20 Hz. Total RNA was then extracted from the lysate using an RNeasy Mini Kit (Qiagen) and included an on-column DNase digestion step. RNA integrity was assessed on the 4200 TapeStation using the RNA ScreenTape assay (Agilent, Santa Clara, USA).

### Library construction and sequencing

RNA samples (*n* = 3) with an RNA Integrity Number above 8.0 were used for library preparation with the TruSeq RNA Sample Prep Kit v2 (Illumina, San Diego, USA) using 500 ng total RNA input. Library quality control was performed on the 4200 TapeStation with the D1000 ScreenTape kit (Agilent). cDNA libraries (*n* = 3) were sequenced on one lane of an Illumina NextSeq550 instrument utilizing paired-end 150-bp reads. Sequencing was performed at the Vienna BioCenter NGS Unit, Vienna, Austria.

### Transcriptome assembly

The raw sequencing reads were subjected to adapter trimming and quality filtering using CLC Genomics Workbench 12 software (Qiagen Bioinformatics, Aarhus, Denmark). Low quality bases (Phred score ≤ 30) and reads shorter than 50 nt were removed. First, the filtered reads from zooid samples (*n* = 3) were used for de novo transcriptome assembly in CLC Workbench, which was based on the *de Bruijn graphs* approach (Zerbino & Birney, 2008) using the assembly parameters: *k-mer* = 35 and bubble size = 300. The parameters have been adjusted based on the assembly output parameters (low number of contigs, high N50, high average contig length and high percentage of mapped reads). The cluster software cd-hit-est with a sequence identity threshold of 0.95 was used to reduce the redundancy of the assembly (Huang et al., 2010). The filtered reads were mapped back to the assembled contigs using the default mapping parameter of the CLC RNA-seq tool (mismatch cost 2, insertion cost 3, deletion cost 3, length fraction 0.5 and similarity fraction 0.8). Read counts were converted to transcripts per million (TPM) for normalization (Wagner, Kin & Lynch, 2012). Analysis of similarity of the three zooid samples was used to test the significance of the clusters revealed by Principal component analysis on the CLC Genomics Workbench 12 software. *Z*-score normalization was used to convert expression values to a common scale. For comparison, de novo transcriptome assembly was additionally performed separately for each replicate as described above. Additionally, global Pearson’s correlation was analysed between samples for TPM normalized values using *ggcorrplot* package (Kassambara, 2019).

### Functional annotation

Functional annotation of the assembled transcripts was performed based on the similarity of the translated nucleotide sequences with proteins in the NCBI protein database, which contains non-redundant GenBank translations together with sequences from other databases (Pruitt, Tatusova & Maglott, 2005). The final list of non-redundant contigs with a consensus length ≥ 300 nt (*n* = 85, 532) were used for functional annotation with the software Blast2GO (Conesa et al., 2005). The contigs were annotated by BLASTX analysis against the NCBI non-redudant protein database. Based on the BLASTX results, annotations were added in Blast2GO using an *E*-value hit filter of ≤ 1.0E–4. The *E*-value distribution for annotated contigs is added in Data S1. To monitor for contaminating gut contents, we mapped assembled contigs against the algae genomes of *Synechococcus elongates* and *Cryptomonas paramecium*, which were the most closely related species with available genome data to the food sources of *F. sultana*.

### KEGG orthology assignment

To validate the annotations assigned by Blast2GO, *F. sultana* transcripts were blasted against the Kyoto Encyclopedia of Genes and Genomes (KEGG) database (Kanehisa et al., 2017) using the KEGG Automatic Annotation Server (KAAS; https://www.genome.jp/tools/kaas/) (Moriya et al., 2007). KEGG orthology (KO) assignment was done using the single-directional best hit (SBH) method for partial genomic data using a threshold greater than 60, against the datasets of 28 related species, which were selected as reference organisms (Table S1).

### Gene ontology

Gene Ontology (GO) IDs of the GO domains: biological process, molecular function and cellular component were separately assigned to the contigs and subsequently merged into higher-order GO terms using the GO slim routine in Blast2GO (Conesa et al., 2005).

## Results

### De novo assembly of *F. sultana* transcriptome

**Figure 2:**
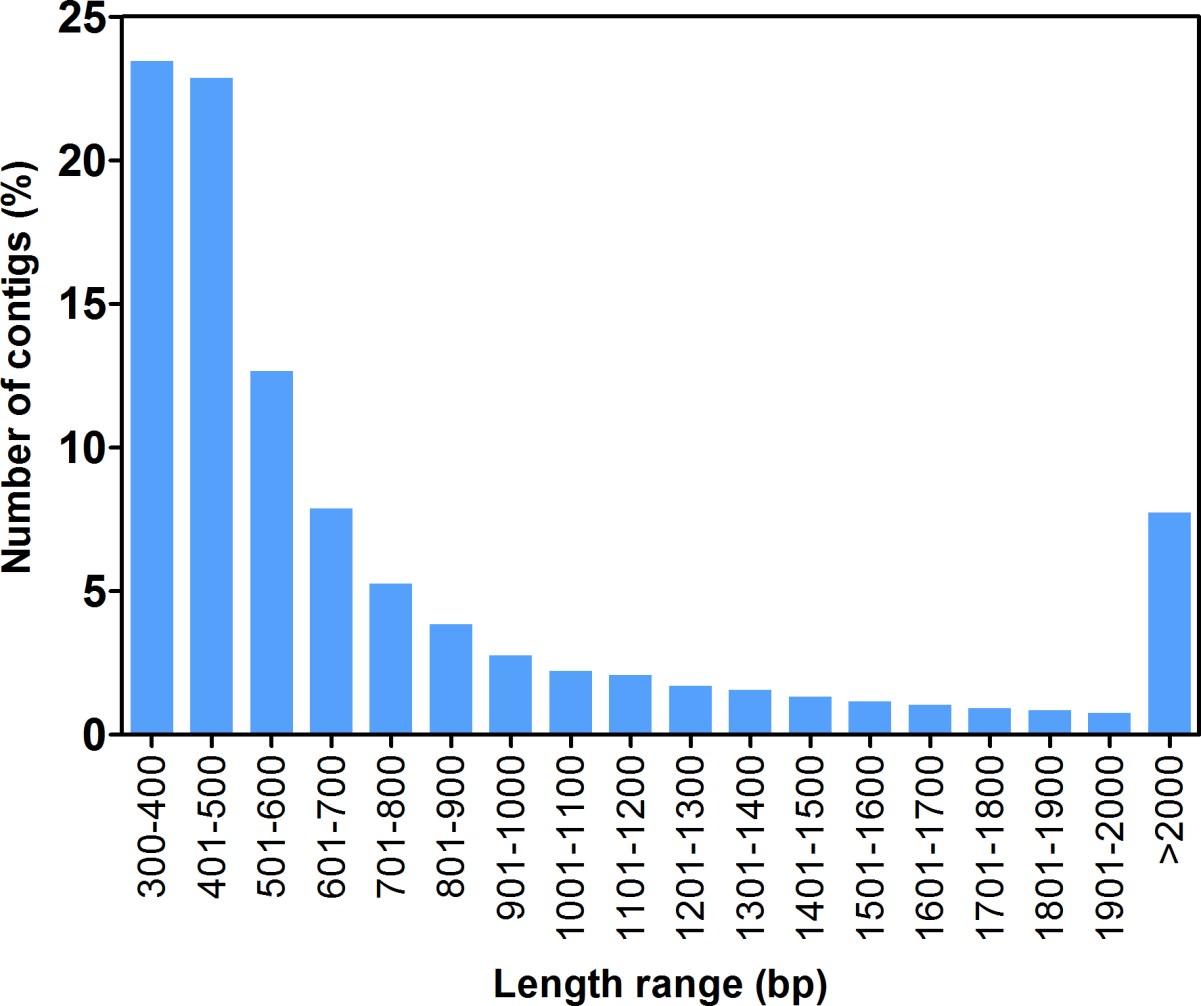
Length distribution of *F. sultana.* transcripts. Sequence length distribution of transcripts assembled from Illumina reads for the bryozoan transcriptome of *F. sultana*. The *x*-axis indicates number of contigs, and the *y*-axis indicates number of transcripts for each size.

We obtained a total of 131 million raw reads (paired) of the *F. sultana* transcriptome. After removal of low-quality bases and contaminating adapter sequences, we obtained a total of 118 million (90.52% of raw reads) high-quality reads (paired) ranging from 37 to 42 million trimmed reads for each of the three libraries (Table 1). De novo assembly of the high-quality reads obtained from all samples was done in CLC Genomics Workbench with different assembly parameters. The default settings of the software and different combinations of *k-mer* (20, 25, 30, 35, and 40) and bubble size (200, 250, 300, 350, and 400) were employed. The final assembly using the parameters, *k-mer* = 35 and bubble size = 300, resulted in the highest N50, the highest average contig length and a similar percentage of reads that could be mapped back to the assembly. The removal of redundant sequences resulted in a total of 85,532 contigs with an average length of 852 bp. An overview of the sequencing and assembly statistics of *F. sultana* zooid is presented in Table 1. The majority of contigs was distributed within a size range of 300 bp to 500 bp and 7.7% of the contigs were larger than 2,000 bp. The lengths of the assembled contigs are represented as a bar chart (Fig. 2).

**Table 1:** Summary statistics of the transcriptome assembly for *F. sultana* using the combined data of three replicate zooid samples. Transcriptome assembly statistics were generated using CLC Genomics Workbench 12 software. Reads used for the de novo assembly were trimmed for Illumina adapters and quality filtered.

**Transcriptome feature**	**Values**
Raw reads (paired)	131,086,668
Paired Reads after quality control	118,644,165
Assembled contigs	86,759
Contigs after redundancy filtration	85,532
Average length (bp)	852
N50	1,085
GC content	51.4%
Trimmed reads mapped to contigs	106,867,585 (90.1%)
Contigs with BLASTX hits	30,488 (35.6%)
Annotated contigs	23,686 (28%)

### Comparison of replicate zooid samples

**Figure 3:**
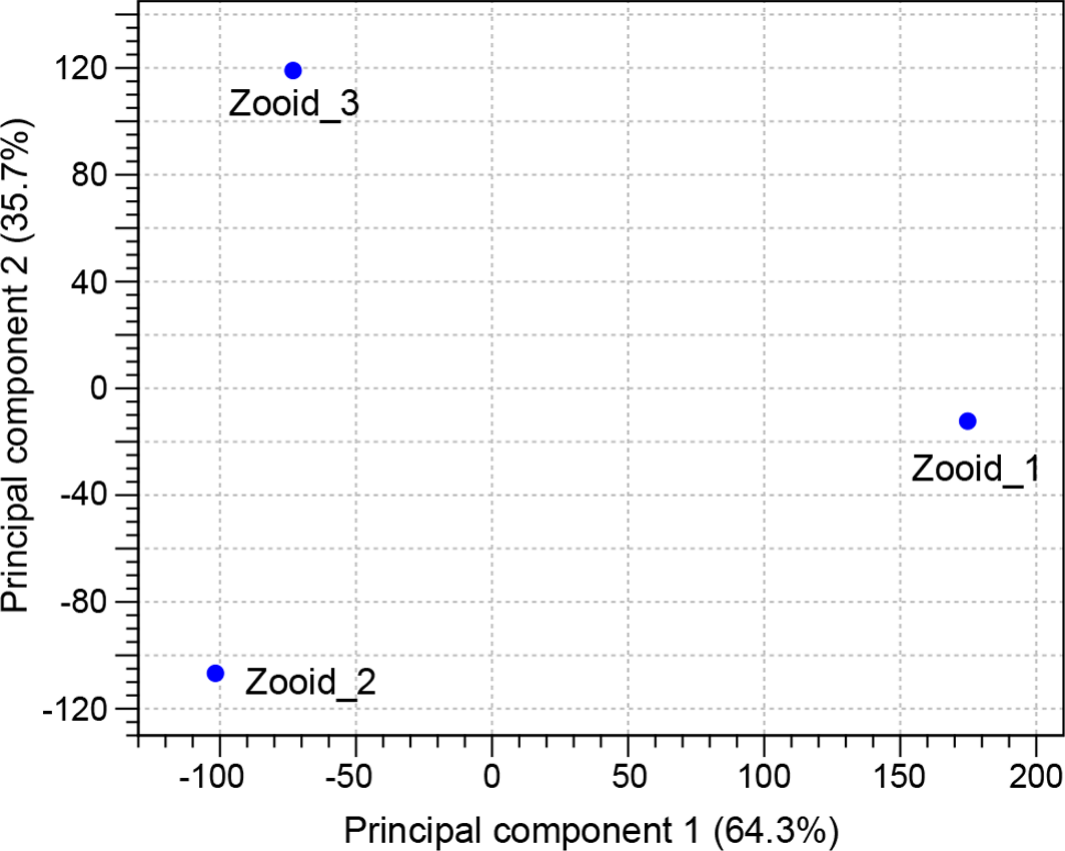
Principal component analysis. PCA plot of normalized RNA-seq expression values of three replicate zooid samples.

**Figure 4:**
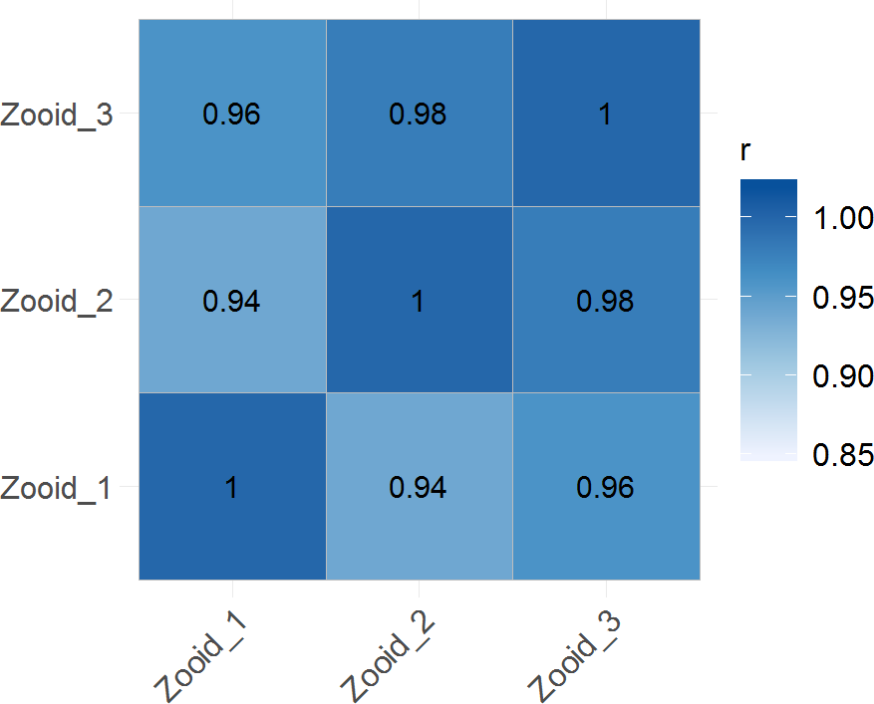
Global correlation analysis. Heatmap showing Pearson’s correlation coefficient (r) for TPM normalized values across samples, indicating positive correlation between biological replicates.

**Figure 5:**
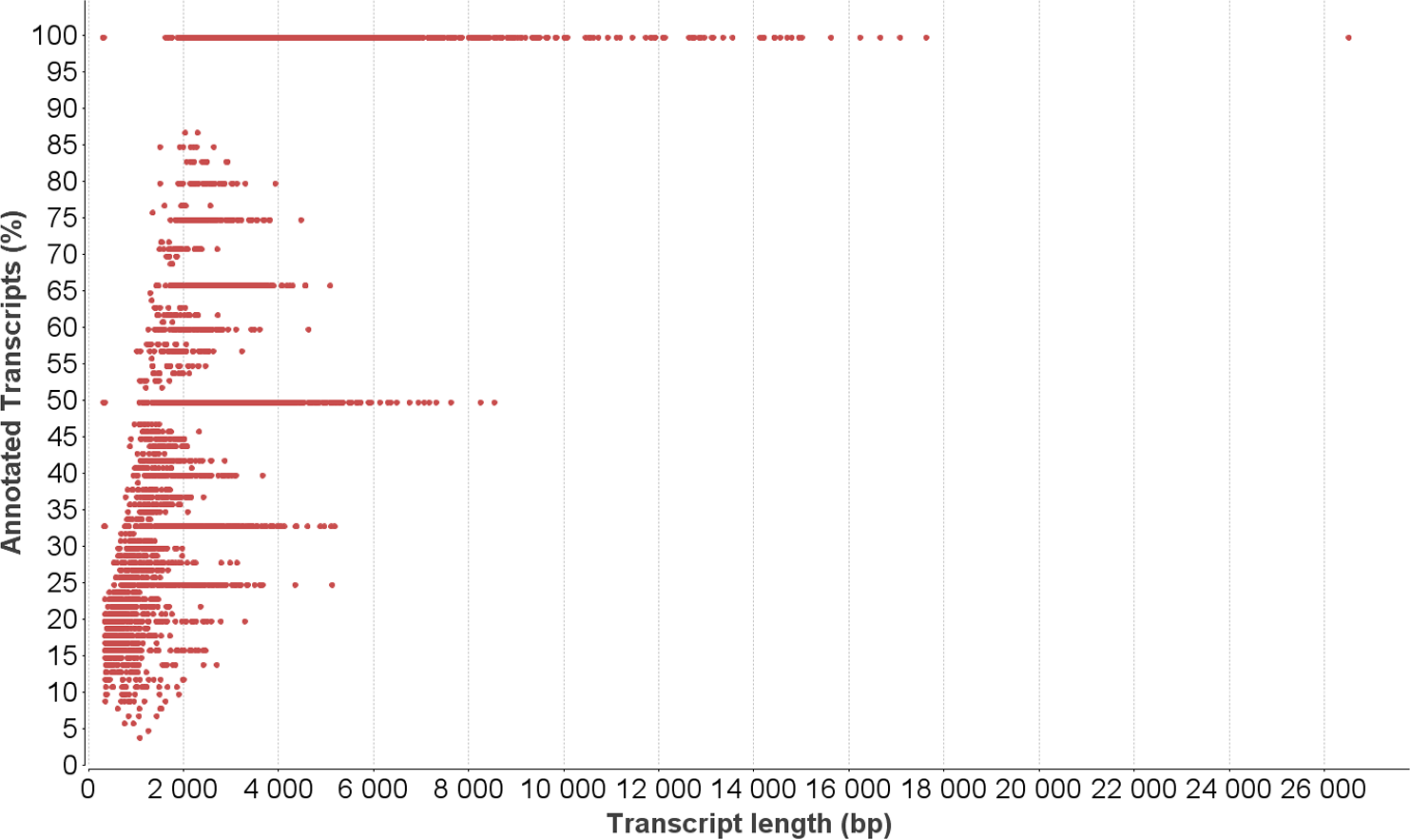
Relationship between functional annotation and transcript length. Percentage of successfully annotated transcripts compared to contig length.

The uniformity of the three zooids was assessed by generating assemblies for each of the replicates alone in addition to the joint assembly of all three samples. Description of sequencing data and assembly statistics of the replicate samples are enclosed in Data S2. The number of non-redundant contigs of the replicate assemblies were 90,145 (zooid 1), 80,634 (zooid 2), and 72,740 (zooid 3). The differences can be explained by differing total read numbers obtained for each of the three samples. The numbers of reads mapped back to contigs resulted in >90% mapped reads for each sample, for replicate and joint assembly (Data S2). Based on the random clustering of the three replicates in the PCA plot (Fig. 3), we assume a high degree of uniformity among the replicates. The correlation between TPM normalized values from replicate samples showed positive correlation between the samples (Fig. 4). When comparing the assembly statistics of the combined and replicate transcriptomes, a high N50 and higher average contig length were obtained for the combined dataset. Longer contigs were especially desired as the size increases the likeliness to annotate the contigs in down-stream BLASTX analysis (Fig. 5). Thus, we decided to use the combined transcriptome from all three zooids for further analysis. Total read counts and normalized expression values (TPM) for the final joint assembly are enclosed in Table S2. Mapping of the assembled contigs did not result in any hits for both algae species.

### Functional annotation

At least 35% (30,488 contigs) of the contigs showed significant similarities in the BLASTX analysis. Annotations for a specific protein could be added to 28% (23,976 contigs) of the transcripts (Table 1). An overview of annotated contigs with all the details is presented in Table S3. The percentage of successfully annotated contigs was increased up to 100% with a larger transcript length (>17,000 bp) and was positively correlated with the transcript length (Fig. 5).

**Figure 6:**
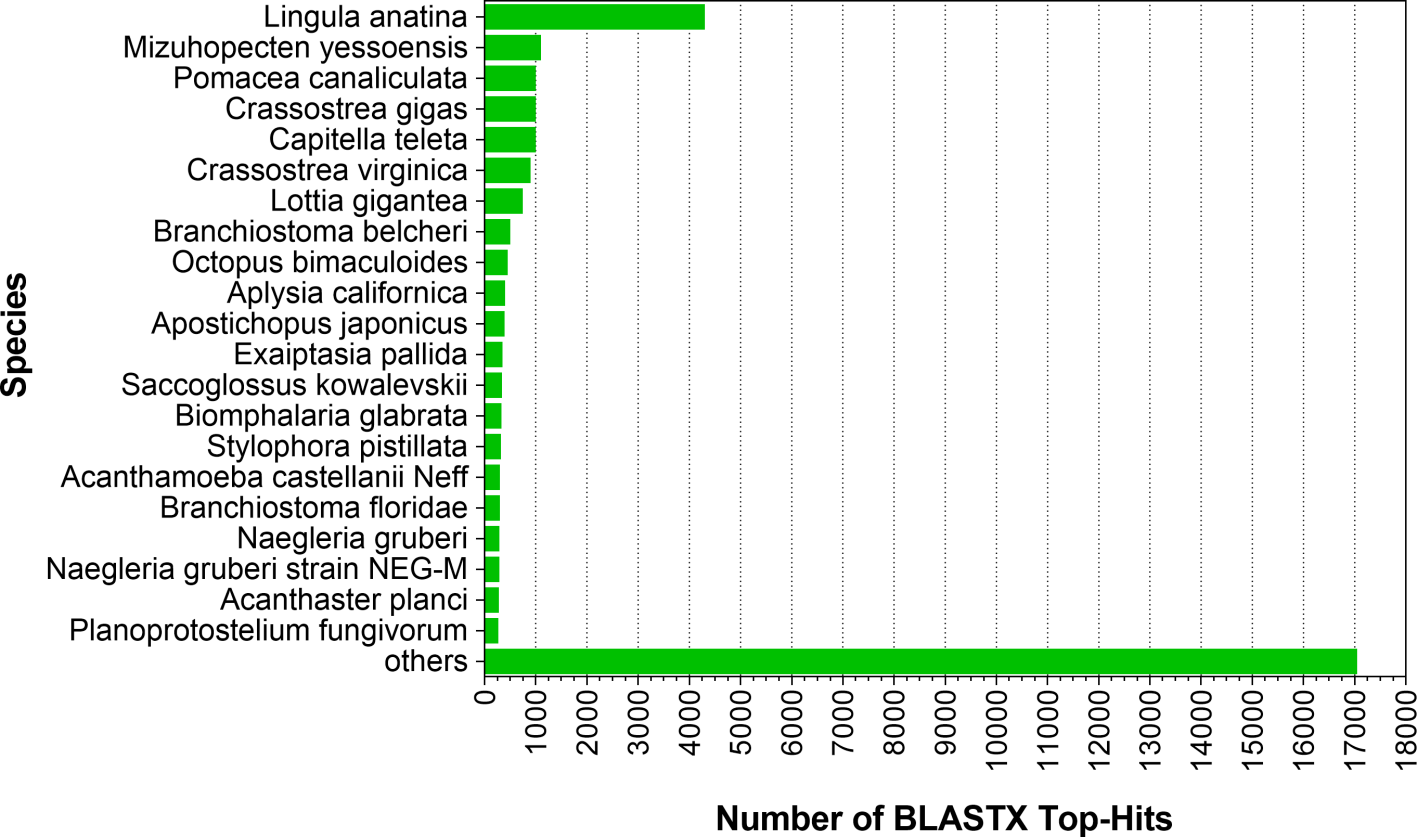
Species distribution of BLASTX hits. Number of hits for the most represented species from BLASTX analysis against non-redundant protein database considering the species with the most significant hit for each transcript.

As the RefSeq protein database covers all species with the relevant data available, the BLASTX results were subdivided according to the species, which resulted in the most hits with the lowest *E*-values per contig. BLAST top hits retrieved from the NCBI database showed only 21 top hits with similarity to *F. sultana* sequences (Fig. 6). The highest numbers of hits to a single species (>4,400) for the *F. sultana* transcripts were derived from the brachiopod species *Lingula anatina*, followed by *Mizuhopecten yessoensis* (yesso scallop) and *Pomacea canaliculata* (golden apple snail) (Fig. 6). The higher similarity to brachiopod species sequences may reflection of the phylogenetic relatedness of these species to *F. sultana*. More than 17,000 transcripts showed similarities with other different species (Fig. 6). As only limited genomic data is available for bryozoans, it can be expected that BLASTX hits are distributed among a wide range of different species.

### KEGG orthology

**Figure 7:**
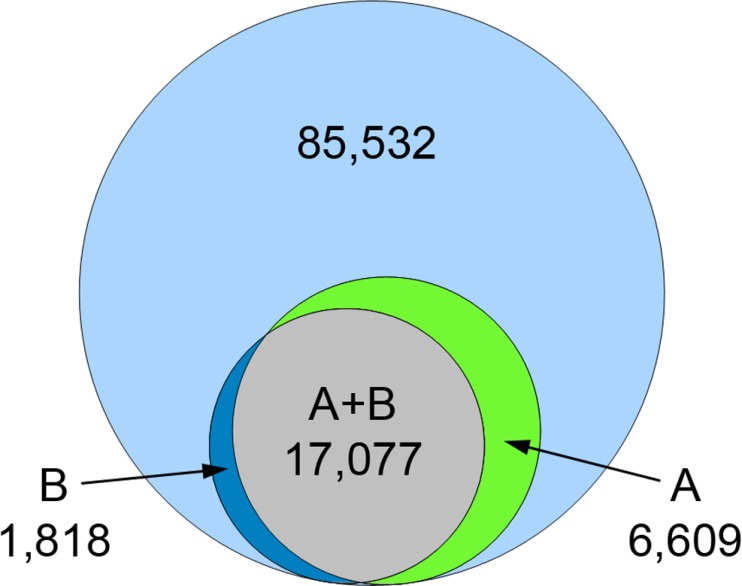
Venn diagram for annotated contigs from Blast2GO and contigs with KEGG orthology (KO) assignment. Overlap of annotated contigs through Blast2GO (A) with contigs that were assigned with KO terms (B), relative to all non-redundant contigs (*n* = 85, 532; outer blue circle).

The assignment of KEGG orthology (KO) terms was used as an alternative approach to confirm the annotations obtained by Blast2GO. Out of 85,532 contigs, KO terms could be assigned to 18,895 transcripts using 28 selected reference organisms. Out of the contigs with KO hits, 17,077 contigs overlap with those 23,686 contigs that were annotated by Blast2GO (Fig. 7). The assigned KO hits could be associated to a total of 388 pathways in the KEGG database. The complete list of KO assignments to annotated *F. sultana* contigs and KEGG pathways is enclosed in Table S1.

### Gene ontology

**Figure 8:**
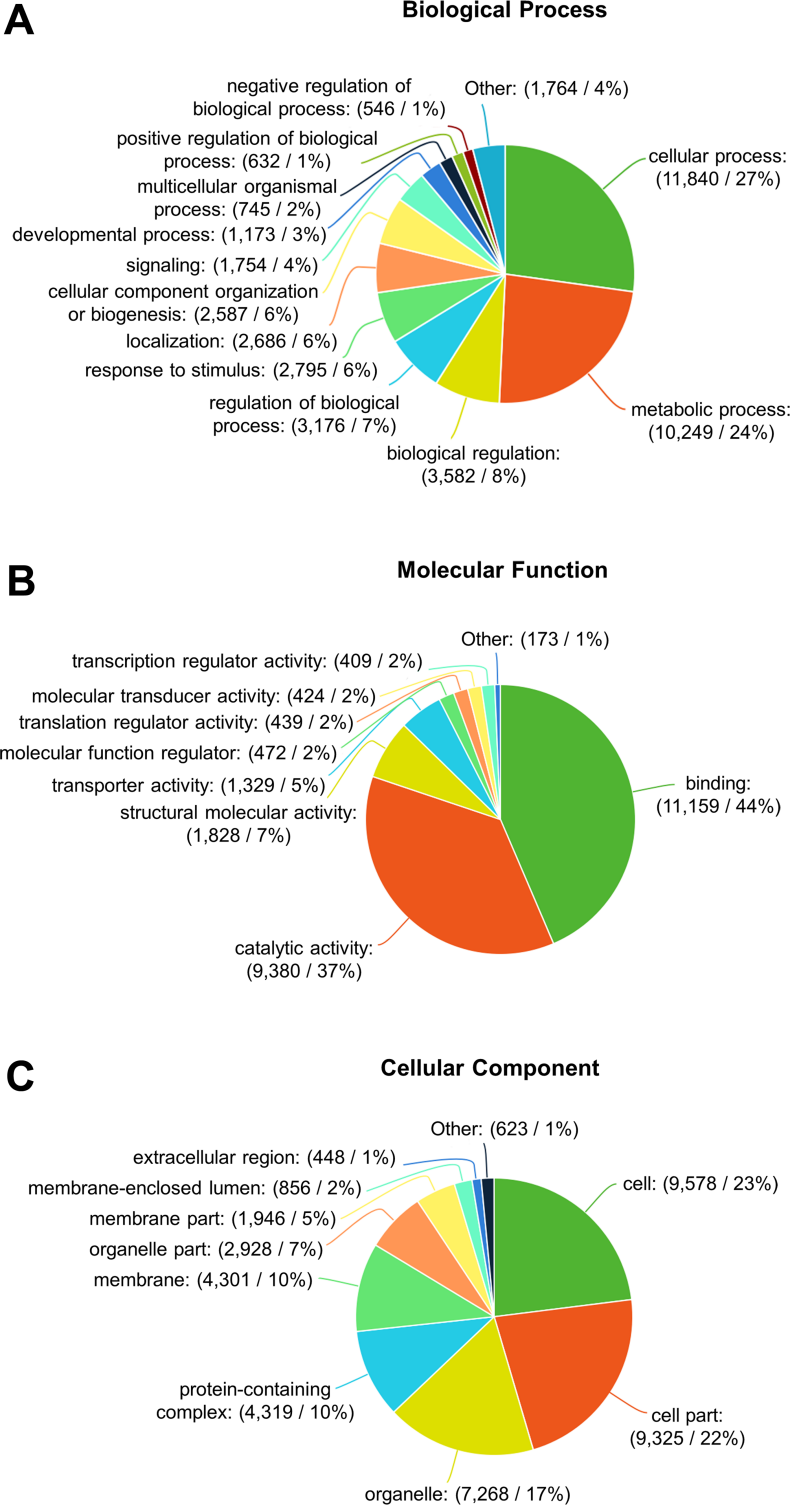
Gene ontology annotation of *F. sultana* transcripts. Frequency of level two GO slim terms in *F. sultana* transcripts under the GO domains, biological process (A), molecular function (B) and cellular component (C).

**Figure 9:**
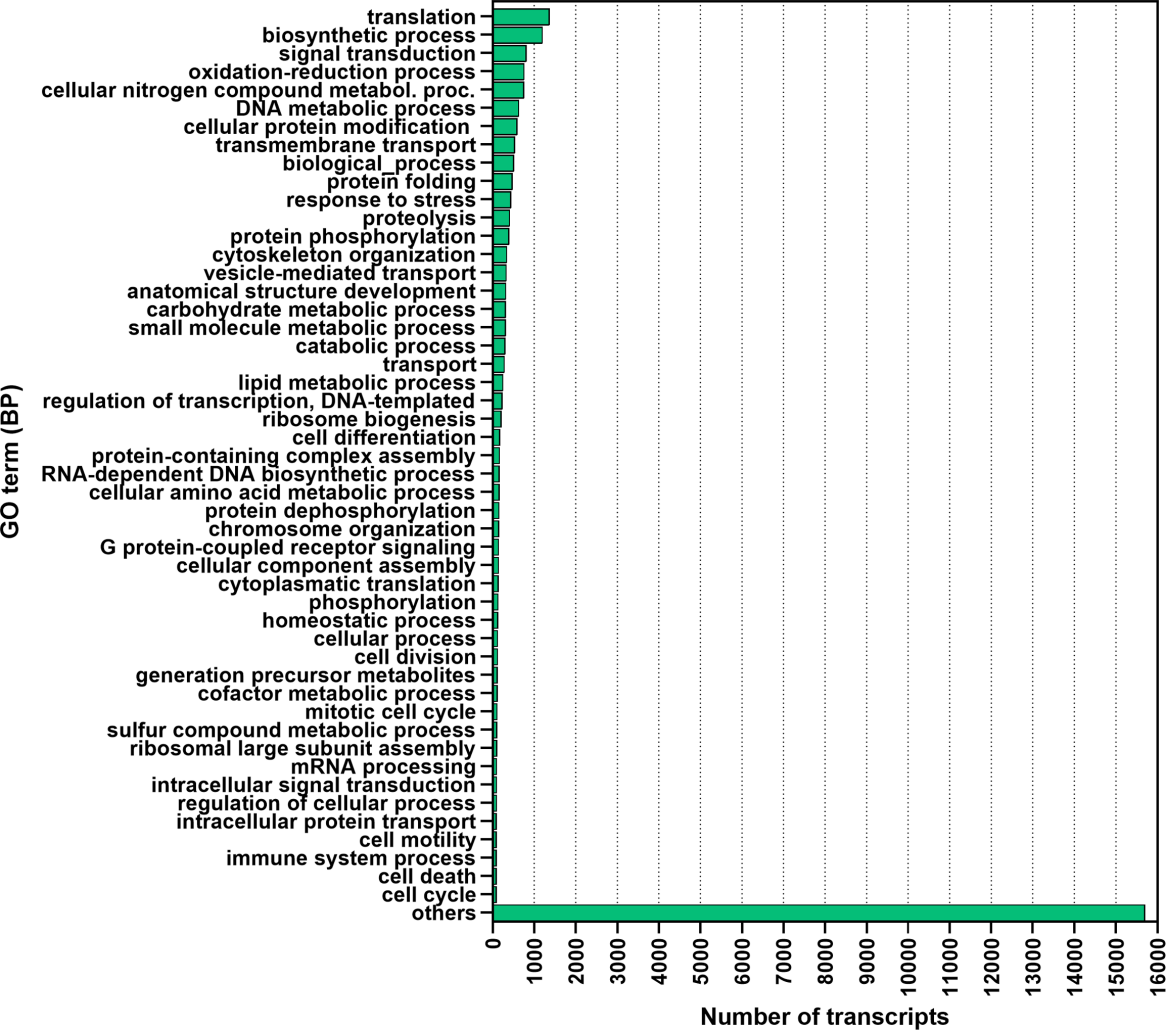
Biological classification of top 50 *F. sultana* transcripts. The analysis of**** biological process of *F. sultana* transcripts was performed at level 3 using Blast2GO.

Based on GO annotation, transcripts were assigned into three GO domains: biological process (43,529), molecular function (25,614), and cellular component (41,592) (Fig. 8). Among the biological process terms, cellular processes (27%) and metabolic processes (24%) were most represented on GO level 2. Further analysis of biological process on GO level 3, showed most transcripts were associated with translation, biosynthesis process, signal transduction, oxidation–reduction process, DNA metabolic process, transmembrane transport, protein folding, response to stress, and proteolysis (Fig. 9). The most represented terms for molecular function were binding (44%) and catalytic activity (37%). Under the cellular component GO term: cell (23%) was most abundant followed by cell part (22%) and organelle (17%) (Fig. 8).

## Discussion

The bryozoan *F. sultana* is the primary host of myxozoan parasite *T. bryosalmonae*; however, the lack of full-genome and transcriptome has hindered the investigation of biology and molecular mechanisms concerning parasite transmission. We performed zooids transcriptome sequencing and reported de novo assembly of *F. sultana*. The *de novo* assembly of *F. sultana* resulted, 85,532 contigs with an N50 of 852 bp and 51.4% of GC content. Only 21 BLAST top hits for *F. sultana* were identified (Fig. 6). The majority of BLAST hits were to mollusk, polychaeate, oyster, snail species*,* that have had a plenty of genomic data generated for them and so hits to these species are much more likely, as found in other invertebrate transcriptome characterization studies. These results indicated a shortage of molecular information on bryozoans in the NCBI database. This *F. sultana* transcriptome assembly may represent a noteworthy proportion of the functional genes in freshwater bryozoans.

Functional annotations using Blast2GO were added to 23,686 contigs. KO assignments were obtained for 18,895 contigs (Fig. 7). Given the larger reference database: NCBI non-redundant protein database (Blast2GO) vs. selected reference datasets (KO) and the more specific blast-algorithm: six-frame translation (Blast2GO) vs. single-directional best hit (KO), we consider the Blast2GO results as the more comprehensive dataset. However, KO assignments were overall in a good agreement with BLASTX annotations, even when using only a limited list of KO reference organisms. The 388 KEGG pathways associated with the KO terms represent a wide range of different physiological and pathological processes. Of particular interest is the high number of transcripts (128) that were assigned to thermogenesis (KEGG pathway identifier: 04714) (Table S1). All genes associated to thermogenesis (reference pathway map from KEGG database) were also found in the *F. sultana* transcriptome. Thermal tolerance activity of marine bryozoans (*Myriapora truncata* and *Pentapora fascialis*) has been studied to understand the response of bryozoans to warmer conditions at the necrosis, growth, respiration structural and mineralogical analysis (Pagès-Escolà et al., 2018). This suggests that *F. sultana* might response to heat and cold stress conditions, and might alter physiological activities through adverse weather conditions.

Functional analysis of the transcriptome annotated and classified the contigs into the GO categories: biological processes, molecular functions and cellular components. GO assignment result of *F. sultana* is similar to the transcriptome of the marine bryozoan *B. neritina*, in which metabolic, reproduction, growth, locomotion, response to stimulus, catalytic and transporter processes were the main groups (Wong et al., 2014). GO annotations revealed that highly abundant genes in *F. sultana* encoded diverse metabolic, transporter, and enzymatic proteins. Additionally, muscle-related genes (actin adductor muscle, tubulin (alpha and beta chains), and myosin (light chain)) and ribosomal proteins (40S ribosomal proteins (S2-S29) and 60S ribosomal proteins (P0 and L3-L39)) were highly abundant in the zooid. Transcripts of myosin, which are the main regulator of muscle growth and development in invertebrates, were found in the bryozoans (Fuchs, Martindale & Hejnol, 2011). Morphological analysis of the muscular ground pattern of bryozoans also suggest an involvement of muscles in the tentacle, lophophoral arms, and epistome form the body wall musculature (Gruhl, 2008; Gawin, Wanninger & Schwaha, 2017). A summary of the top most 120 abundant contigs based on TPM expression values are presented in Table S4.

We found many calcium-binding (mammalian ependymin-related protein 1, calcineurin subunit B type 1, agrin-like isoform X2, and calmodulin) and extracellular matrix proteins (insoluble matrix shell protein 5 and extracellular matrix protein FRAS1-like) in the *F. sultana* zooid (Table S3). Those proteins have also been reported to take part in body formation (Qian, Wong & Zhang, 2010; Brown, 2011). Additionally, we also identified that homeobox protein Hox-A5-like, tyrosinase, perlucin, chitinase, mucin, Von Willebrand factor type A protein are common body formation-associated constituents shared by invertebrates, implying that this set of essential genes were used by their common ancestor.

Identification of transcripts related to proteolysis is of interest for many invertebrate aquatic animals like bryozoan because of the role of these enzymes play in defense. Transcripts of genes with putative proteolysis functions (GO: 0006508) were found in the transcriptome of *F. sultana* (Table S5). Among them, genes encoding different groups of proteolysis factors and their involvement in protein degradation were identified, such as serine protease, probable serine carboxypeptidase, carboxypeptidase (B, D, and E), peptidase, dipeptidase, cysteine protease, matrix metalloproteinase and cathepsin (B and L). This suggests that exo- and endopeptidases participate in physiological and cellular processes in the zooids. Interestingly, several transcripts encoding for lysosomal signaling were also identified, such as lysosomal protective protein, lysosomal Pro-X carboxypeptidase, lysosomal aspartic protease, and lysosomal trafficking regulators.

The identification of transcripts involved in response to stress and immune function are of interest because of the bryozoan’s role as primary host of *T. bryosalmonae*. The GO annotation identified 450 transcripts (Table S5) that are potentially related to stimulus responses (GO: 0006950). The heat shock protein family playing an important role in thermal tolerance (Garbuz & Evgen’ev, 2017) were the most abundant transcripts in this category. Heat shock genes are necessary for protein folding, multimer dissociation and association, translocation of proteins across membranes, and regulation for adaptation of organisms to a harsh environment (Garbuz & Evgen’ev, 2017). Since *F. sultana* is a eurythermal species with great tolerance of cold water (Økland & Økland, 2001), abundant of heat shock proteins could support more efficient folding of proteins at low temperatures. Additionally, GO analysis identifed 102 transcripts (Table S5) that are classified as immune system (GO: 0002376). Several transcripts involved in innate and adaptive immunity were also observed. For example, thioredoxin, protein toll-like, toll-like receptors (TLR3, TLR4, TLR7, and TLR13), and c-type lectin domain family 10 member A, are key components of innate and adaptive immunity (Qian, Wong & Zhang, 2010; Satake & Sasaki, 2010). Future studies exploring the roles of immune related genes in this bryozoan would lead to a better understanding of host-parasite interactions. Interestingly, ras-related protein, galactose-specific lectin nattectin, tumor necrosis factor receptor superfamily, and apoptosis regulatory protein including voltage-dependent anion-selective channel protein, were also identified and are involved in pro-inflammatory and apoptosis activities (Qian, Wong & Zhang, 2010).

## Conclusions

We provide here a transcriptome of zooids of *F. sultana*. To our knowledge, our study reports the first transcriptomic analysis of freshwater bryozoans and enriches the public bryozoan transcript databases. This transcriptome provides an overview and preliminary analyses on *F. sultana* and is a valuable starting point for comparative analyses of transcriptomes of other bryozoan species. Furthermore, *F. sultana* transcriptome will help facilitate whole genome sequencing and annotation. The identified and annotated transcripts will provide valuable genomic resources for the understanding of the unique biological characteristics, such as cellular, metabolic, binding, and catalytic processes, of this freshwater bryozoan species. We believe that this transcriptome will also assist as a valuable resource for further study of developmental gene expression, transcriptional regulation, functional genomics, and marker development applications in freshwater bryozoans.

##  Supplemental Information

10.7717/peerj.9027/supp-1 Data S1 *E*-value distribution of annotated contigs by BLASTX Number of annotated contigs relative to the respective *E*-values (cut-off ≤ 1.0E–4) obtained through Blast2GO.

10.7717/peerj.9027/supp-2 Data S2 Description of sequencing data and transcriptome assembly statistics for three replicate zooid samples Transcriptome assembly statistics were generated using CLC Genomics Workbench 12 software. Reads used for the de novo assembly were trimmed for Illumina adapters and quality filtered.

10.7717/peerj.9027/supp-3 Table S1 KEGG orthology (KO) assignment KEGG orthology (KO) assignment. Reference organisms used for KO analysis, list of KO terms assigned to annotated *F. sultana* contigs and list of KEGG pathways associated with assigned KO terms.

10.7717/peerj.9027/supp-4 Table S2 Number of replicate reads mapped back to *F. sultana* contigs Number of replicate reads mapped back to *F. sultana* contigs. Total read number and TPM expression values mapping to each contig for the three replicate zooid samples.

10.7717/peerj.9027/supp-5 Table S3 List of annotated *F. sultana* contigs List of annotated *F. sultana* contigs. This file contains all the information of annotated contigs for zooids through Blast2GO.

10.7717/peerj.9027/supp-6 Table S4 List of top 120 most abundant* F. sultana* contigs List of top 120 most abundant* F. sultana* contigs. This file contains sequence name, description, length, TPM and RPKM expression values, total read counts and GO IDs of top 120 most abundant* F. sultana* contigs.

10.7717/peerj.9027/supp-7 Table S5 List of *F. sultana* transcripts related to proteolysis, response to stress and immune system process List of *F. sultana* transcripts related to proteolysis, response to stress and immune system process. This file contains transcripts associated with the GO terms: proteolysis (GO: 0006508), response to stress (GO: 0006950) and immune system process (GO: 0002376).

## Additional Information and Declarations

### Data Availability

The following information was supplied regarding data availability: The raw reads are available in the NCBI Sequence Read Archive database: SRP198874 and BioProject ID PRJNA543711. The final transcriptome assembly is available from the NCBI Transcriptome Shotgut Assembly database: GHLZ00000000 and Kumar, Gokhlesh; Ertl, Reinhard; Bartholomew, Jerri L.; El-Matbouli, Mansour (2020): F_sultana_all_contigs_copy_v2.fsa. figshare. Dataset. https://doi.org/10.6084/m9.figshare.11627580.v1.
