# Supplementary figures and images for: First transcriptome analysis of bryozoan Fredericella sultana, the primary host of myxozoan parasite Tetracapsuloides bryosalmonae
Source: PeerJ. 2020 Apr 28;8:e9027. doi: 10.7717/peerj.9027 (PMC7194087; doi:10.7717/peerj.9027)

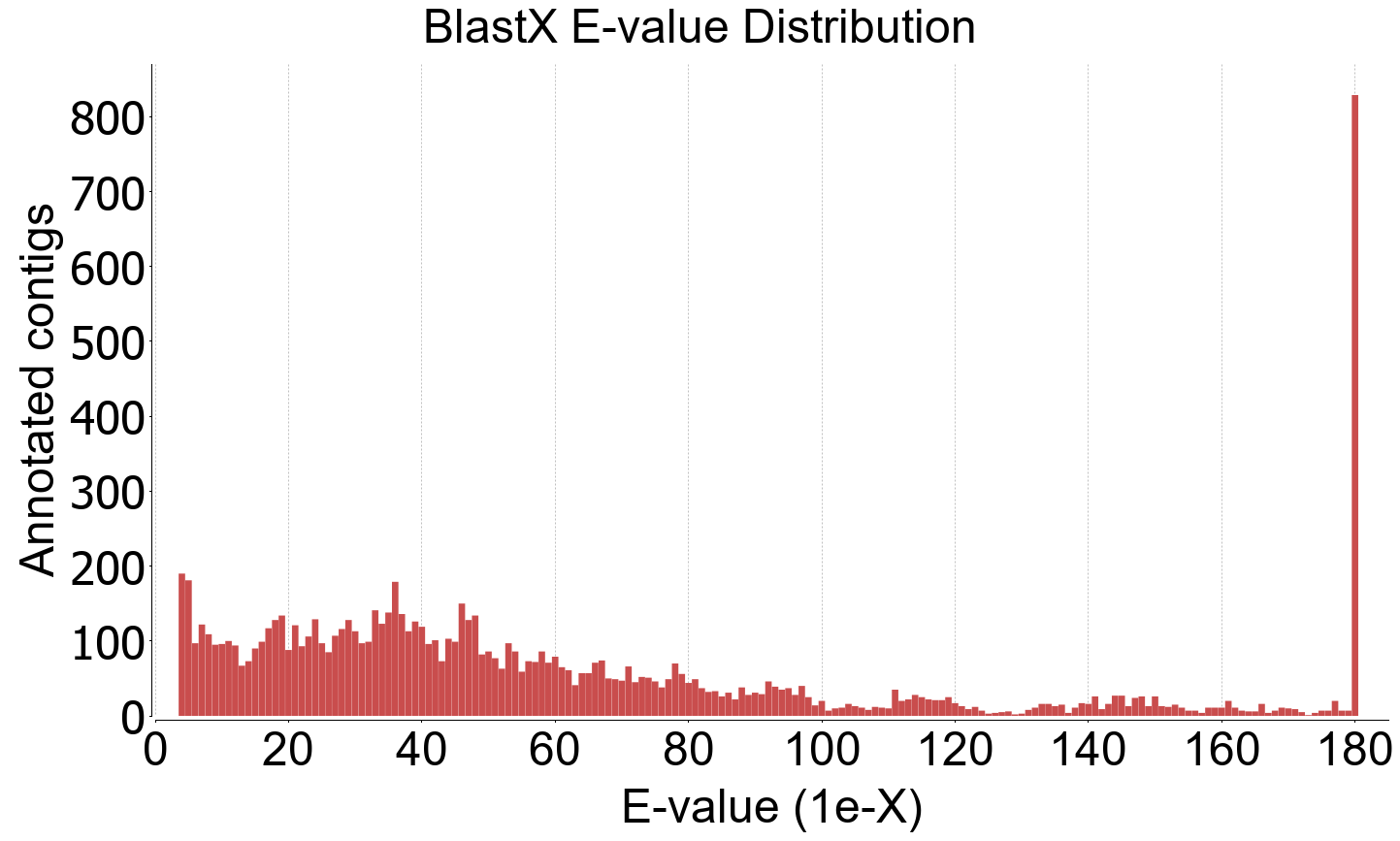

Supplement: Data S1 — Number of annotated contigs relative to the respective E-values (cut-off ≤ 1.0E–4) obtained through Blast2GO. [file peerj-08-9027-s001.png]
