# Supplementary material for: First transcriptome analysis of bryozoan Fredericella sultana, the primary host of myxozoan parasite Tetracapsuloides bryosalmonae
Source: PeerJ. 2020 Apr 28;8:e9027. doi: 10.7717/peerj.9027 (PMC7194087; doi:10.7717/peerj.9027)
Supplement: Data S2 — Transcriptome assembly statistics were generated using CLC Genomics Workbench 12 software. Reads used for the de novo assembly were trimmed for Illumina adapters and quality filtered. [file peerj-08-9027-s002.pdf]

Supplementary Data 1: Description of sequencing data and transcriptome assembly statistics of three replicate zooid samples.

**Transcriptome feature**	**Replicates**		
	**Zooid 1**	**Zooid 2**	**Zooid 3**
Raw reads (paired)	47,040,144	42,691,704	41,354,820
Paired reads after quality control	42,441,722	39,040,613	37,161,830
Duplicated reads	142,699 (0.34%)	119,997 (0.31%)	120,536 (0.32%)
Assembly parameter: *k-mer*/bubble size	35/300	35/300	35/300
Assembled contigs	92,581	83,011	74,811
Contigs after redundancy filtration	90,145	80,634	72,740
Average length (bp)	642	665	690
N50	919	1,015	1,108
GC content	51.8%	52.7%	52.9%
Trimmed reads mapped to respective replicate assembly	38,560,038 (90.8%)	35,722,612 (91.5%)	33,787,617 (90.9%)
Trimmed reads mapped to combined assembly (generated from all three zooid samples)	38,241,072 (90.1%)	35,140,999 (90%)	33,485,514 (90.1%)
